# State of the Art and Future of Stem Cell Therapy in Ischemic Stroke: Why Don’t We Focus on Their Administration?

**DOI:** 10.3390/bioengineering10010118

**Published:** 2023-01-14

**Authors:** Andrea Valeri, Emanuela Mazzon

**Affiliations:** IRCCS Centro Neurolesi “Bonino-Pulejo”, Via Provinciale Palermo, Contrada Casazza, 98124 Messina, Italy

**Keywords:** stem cells therapy, stroke, mechanism of stem cells therapy, route of administration, precondition

## Abstract

Stroke is one of the leading causes of death and disability worldwide, so there is an urgent need to find a therapy for the tragic outcomes of this cerebrovascular disease. Stem cells appeared to be a good solution for many conditions, so different experiments were made to establish stem cells as a feasible therapy for stroke. The aim of this review is to analyze the state of the art of stem cell therapy for stroke and if the route of administration could represent a valid adjusting point for ameliorating the therapy’s outcome. To obtain this, we searched the scientific literature of the last 10 years for relevant in vitro and in vivo evidence regarding stem cells’ potential in stroke therapy. In vitro evidence points to hypoxia, among the preconditioning strategies, as the most used and probably efficient method to enhance cells qualities, while in vivo results raise the question if it is the type of cells or how they are administrated which can make the difference in terms of efficiency. Unfortunately, despite the number of clinical trials, only a few were successfully concluded, demonstrating how urgent the necessity is to translate pre-clinical results into clinics. Since any type of stem cell seems suitable for therapy, the chosen route of administration corresponds to different engraftment rates, distribution and efficiency in terms of the beneficial effects of stem cells. Intravenous administration was widely used for delivering stem cells into the human body, but recently intranasal administration has given promising results in vivo. It allows stem cells to efficiently reach the brain that was precluded to intravenous administration, so it is worth further investigation.

## 1. Introduction

One of the most common neurological conditions which represents a life-threatening event is cerebrovascular disease. In particular, stroke is responsible for more than 5 million cases of long-term disability in currently alive stroke survivors, while it represents the third cause of death [1]. In general, brain stroke occurs when the blood flow to the brain is impaired, and it is considered a serious medical emergency that requires treatment as soon as possible to limit the damage to the brain cells.

Stem cells are a particular type of cell that has attracted the scientific community due to their potential in regenerative medicine. Indeed they can differentiate in a certain variety of cell types, along with possessing self-renewal capacity, so their medical application seems to have virtually no limits [2]. However, the use of stem cells as routine therapy is far to be reached. The road to obtaining complete safety and standardizing administration protocols is not yet at its end, but undoubtedly progress in this field is evident [3].

As will be discussed in the further sections, there are different types of stem cells, each of which has different potential in regenerative medicine. Given the serious condition and social burden of stroke, the scientific community tried to optimize the use of stem cells as a treatment. There are indeed guidelines for stroke prevention, but not all cases can be efficiently prevented. Finding a cure for the survivors and limiting the disability of a stroke outcome is an urgent matter.

The aim of this review is to investigate the efforts of the scientific community in stem cell application in stroke models, both in vitro and in vivo, and analyze the problem from a different point of view. Given the variety of experiments done, administration routes are taken into consideration as a variable that can be adjusted to optimize the therapy.

## 2. Methodology

**Figure 1:**
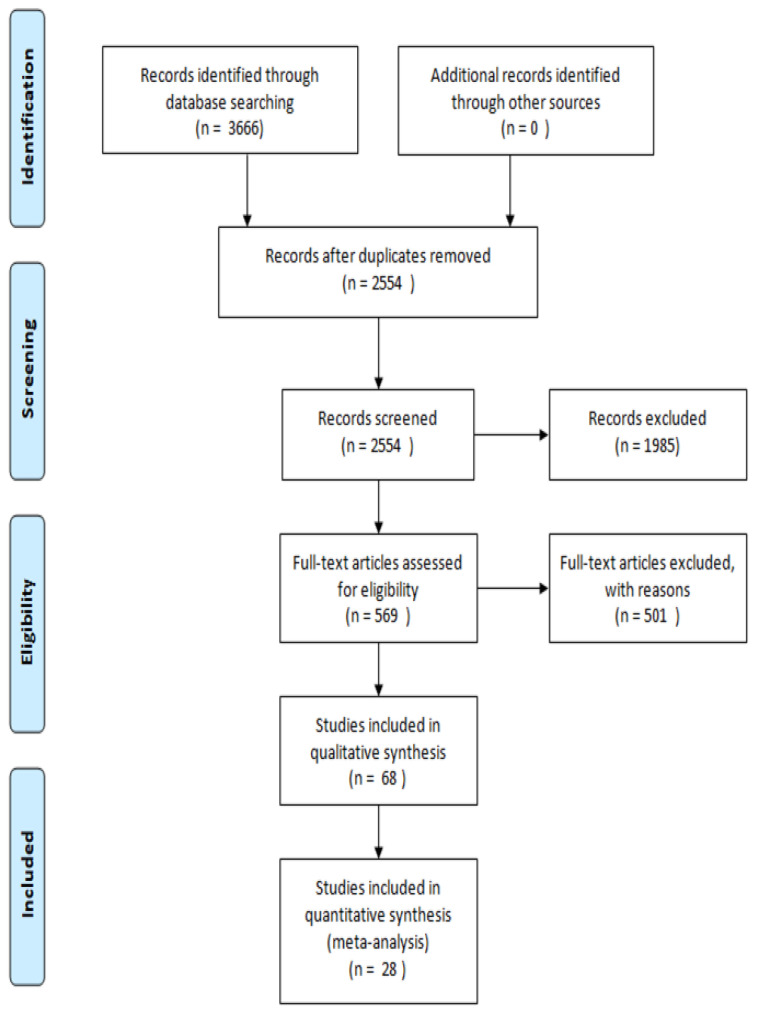
Prisma flow diagram of review article selection.

The publications taken into consideration range from 2012 to 2022. PubMed database was used to retrieve evidence and the keywords used were: “stroke”, “stem cells”, “intraperitoneal”, “intranasal”, “intrathecal”, “intracortical” and “preconditioning”. Reviews and articles not in English were excluded from the analysis, as well as articles not coherent with the topic. Articles regarding in vitro and in vivo experiments that show the effect of stem cells in models of stroke were investigated. The Prisma flow diagram reported in Figure 1 represents the article selection process [4].

## 3. Stroke: Description, Outcomes and Possible Therapies

A stroke is defined as an event that prevents normal blood flow in the brain. The brain is an organ where the blood flow is fundamental because its block and the consequent lack of oxygen and nutrients leads to the death of neural cells within minutes [5]. 

The cases of strokes can be distinguished into ischemic and hemorrhagic strokes. Acute ischemic stroke (AIS) happens when the blood flow to the brain is suddenly interrupted because of the block of one or more blood vessels. Two main parts can be distinguished: a *penumbra* area, where the damage is not yet irreversible, even if the area is not correctly perfused, and a *core* area where, unfortunately, the normal functions cannot be retrieved because the injury is permanent [6]. Neurons continue to consume energy, leading to a rapid depletion of adenosine-triphosphate (ATP) and the start of lactate acidosis, resulting in ionic imbalance. Neurotransmitter release is triggered, and the consequence is excitotoxicity, also thanks to the inhibition of the neurotransmitter reuptake mechanism. Subsequently, the calcium overload of the cells switches on degradation mechanisms, such as the one from phospholipase action and proteases. Moreover, sodium and water flow inside the cells, adding more stress and resulting in cell swelling and edema. The consumed ATP accumulates as adenosine-monophosphate (AMP), which, along with sodium and calcium levels, dramatically increases mitochondrial activity and the consequent reactive oxygen species (ROS) production [7]. Oxidative stress is one of the main causes of neuronal death in the principal neurodegenerative diseases [8], so it represents a source of danger for the neurons that are still alive. Microglia behavior has to be taken into account during ischemic stroke. There is evidence that resident microglia could be helpful during ischemic stroke [9], but one does not have to forget that microglia react to an inflammatory environment [10], which could be the brain when a massive amount of neurons die.

Hemorrhagic stroke, on the other hand, happens after the rupture of a blood vessel and the consequent bleeding. It is intracerebral when the bleeding happens inside the parenchyma of the brain and subarachnoid when the rupture causes the blood to flow in the subarachnoid space. The initial source of damage to the brain is the pressure since the blood causes a mass effect that compresses the brain and causes the mechanical loss of neuronal cells. Subsequently, neurotransmitters are released, resulting in excitotoxicity and mitochondrial dysfunctions. Blood and all its components are free to come in contact with brain cells, normally protected by the blood-brain barrier. The products of coagulation and hemoglobin breakdown activate microglia [11]. The blood and damage-associated molecular patterns (DAMPs) resulting from the injured tissue create the right environment for cytotoxicity, inflammation and oxidative stress. In particular, the erythrocytes get degraded, and the heme inside these cells, as well as iron, concur in the cytotoxicity and the generation of ROS, thanks to the Fenton reaction. The final outcome of all these reactions is neuronal death [12]. 

The outcomes of stroke, as it is predictable, involve the correct functioning of the central nervous system, and, for some people, there is no full recovery [13]. 

Surgical and pharmacological interventions are possible to limit the devastating outcomes of stroke, even though some of them are strictly correlated to the time passing between the stroke event and the actual intervention. Currently, the Food and Drug Administration (FDA) approved the use of recombinant tissue plasminogen activator (t-PA) as an intervention for acute ischemic stroke. However, only a small percentage of patients arrive at the hospital in the right timeframe (4.5 h) for this drug to be efficient [14]. For this reason, efforts were made to extend this timeframe or to research new drugs able to limit brain damage [15]. The restoration of blood flow can be obtained with mechanical thrombectomy, which consists of the removal of a blood clot through a catheter that grasps and removes it. It has to be remembered that there are specific guidelines that define which patient is eligible for this surgical procedure [16].

Several tentative innovations of the present techniques and the development of new ones were developed. New drugs are in development, and one of them is Maraviroc: it hampers the C-C chemokine receptor 5 (CCR5), based on the evidence that its block enhances brain plasticity, as well as improves learning and memory. CCR5, after stroke, increases its expression in neurons, and this expression remains during the recovery. This increased expression in neurons is accompanied by a decreased expression in microglia and based on this imbalance, authors investigated the effects of CCR5 knockdown. The results indicate that CCR5 knockdown improves motor recovery when it is done in the premotor and motor cortex. It is important to mention that patients with the mutation that naturally knocks down this receptor have a better recovery after stroke compared to patients without this mutation [17]. 

Furthermore, Fluoxetine is used for the treatment of mental diseases like depression or obsessive-compulsive disorder; however, the inhibitors of serotonin uptake seemed to also be efficient in functional recovery after stroke. Unfortunately, it seems that Fluoxetine can only give an improvement in motor functions and, on the other hand, it increases the risk of fractures, seizures and hyponatremia. Probably due to its primary use in mental diseases, the ratio of depression was decreased in treated patients compared to the control group. In addition, it might be useful for patients with mood dysfunctions but does not seem to be indicated for wide use [18]. Besides drugs and surgery, rehabilitation is also important for recovery. Activity-based therapies were found to be beneficial because they represent a trigger for neural plasticity. However, these have to be intended as an add-on since it is unlikely that physical activity alone can resolve all the damages. Some therapies failed to demonstrate their efficacy compared to standard care, revealing once again the huge variability between the different stroke cases. It is important to mention the limitations of some studies. The wide timeline for eligibility sometimes included heterogeneous populations, with different outcomes related to the time post-injury, or some patients also have different deficits, so there could not be any kind of standardization between the different cases [19].

The capacity of the stems for self-renewal and to differentiate in a large variety of mature cells makes them appealing for potential stroke therapy. Initially, scientists thought that the stem cells would simply differentiate into new brain cells and replace the dead ones, but the studies based on this approach did not deliver consistent results [20]. The current benefit of stem cells is thought to be the release of trophic factors that trigger the repair mechanism and stimulate plasticity, like Brain-Derived Neurotrophic Factor (BDNF) or vascular endothelial growth factor (VEGF). It is also important to mention the neuroprotective action of stem cells, which is crucial in preserving the existent cells from apoptosis [21]. The immunomodulatory capacity of stem cells, in particular mesenchymal stem cells (MSCs), is also reflected in the influence that they have on the immune system. Regarding the cells in the brain environment, MSCs shift microglia type from M1 to M2 so they can contribute to the repair of damaged brain tissue. Other cells from the immune system are influenced by MSCs. Neutrophils see a reduction in their accumulation thanks to MSC administration, which also contributes to enhancing the beneficial effects of neutrophils triggered by IL-17 production by CD4-positive T cells. A similar capacity is also replicated for CD4 and CD8 positive cells, as well as for dendritic cells. The effect on the B cells resulted in an increase in IL-10 production [22].

## 4. Stem Cells: Definition, Types and Characteristics

Before discussing the potential applications of stem cells in stroke therapy, it is important to define what exactly a stem cell is.

A stem cell is defined as an undifferentiated, self-renewable cell that possesses the capacity to differentiate into another cell type. It is totipotent when it can give rise to an entire organism. This is the property of cells that derive from the zygote stage until the morula (eight cells) stage. When the cell is not totipotent anymore but retains the capacity to generate endoderm, mesoderm and ectoderm, it is defined as pluripotent. The multipotent cell still retains differentiating potential, but the possibilities are limited compared to the previous ones since they are able to differentiate only into their specific lineage [23].

According to the developmental stage, stem cells could be adult or embryonic [24].

A further division classifies stem cells according to their origin and clinical application. The first point that has to be clear when talking about embryonic stem cells (ESCs) is that they are not totipotent. They are actually pluripotent because they derive from the inner cell mass of the pre-implantation blastocyst, so the stage mentioned before to be classified as totipotent is already gone [25]. However, mainly thanks to three transcription factors (Octamer-binding transcription factor 4, OCT4; SRY-Box Transcription Factor 2, SOX2; Nanog Homeobox, NANOG), they retain the capacity to form the three embryonic layers [26]. Every tissue of the body possesses a certain degree of regeneration. Tissue-specific progenitor stem cells (TSPSCs) are important because they maintain the homeostasis of tissue, and they replace old and dying cells as part of the normal turnover. To prevent the rise of tumors during cell replacement, their self-renewal capacity is strictly controlled in order to ensure that the differentiation is accompanied by a reduction in proliferation [27]. Probably one of the most famous and widely studied types of stem cells, mesenchymal stem/stromal cells (MSCs) express specific marker sets (Endoglin, CD105; ecto-5′-nucleotidase, CD73; Cluster of Differentiation 90, CD90) and must not express another marker set (protein tyrosine phosphatase, receptor type C, CD45; Sialomucin, CD34; Cluster of Differentiation 14, CD14; Cluster of Differentiation 11, CD11b; B-cell antigen receptor complex-associated protein alpha chain, CD79alpha; Cluster of Differentiation 19, CD19; Human Leukocyte Antigen–DR isotype, HLA-DR), as defined by the International Society for Cellular Therapy. It also imposes that MSCs must differentiate into osteoblasts, adipocytes and chondroblasts in vitro, while they have to adhere to plastic when maintained in standard culture conditions [28]. The umbilical cord stem cells (UCSCs) can be harvested after birth and is safe for both baby and mother. The collection stands from days to weeks, and they have the important advantage of reducing graft-vs-host disease, thanks to a better tolerance to Human Leukocyte Antigen mismatch. Sub-populations of stem cells can be isolated from UCSCs. Hematopoietic stem cells, endothelial progenitors, MSCs, epithelial stem cells and induced pluripotent stem cells can be generated [29]. The bone marrow’s main task is to provide a source of hematopoiesis, and it is mainly composed of hematopoietic stem cells, which give rise to blood cells and stromal cells, responsible for the production of fat, bones and cartilage. Initially, their use was limited to cancer treatment, but nowadays, new prospects open for dental diseases, diaphragm dysfunctions and liver diseases. Together, they are defined as bone marrow stem cells (BMSCs) [30]. Probably one of the greatest medical discoveries in recent years, which granted the Nobel Prize in Medicine in 2012 to Sir John B. Gurdon and Shinya Yamanaka, is induced pluripotent stem cells (iPSCs). These somatic cells are reprogrammed in order to return to an embryonic-like state. Oct3/4, Sox2, c-myelocytomatosis oncogene product (c-Myc) and Kruppel-like factor 4 (Klf4) appeared to be essential factors for the generation of iPSCs [31], while later c-Myc was revealed to be not essential [32]. The characteristic of being pluripotent allowed the scientists to overcome the ethical limitation of ESCs use, especially human ones.

## 5. In Vitro Preconditioning: Enhancing Capacities for Future Applications

Stem cells possess a large variety of beneficial properties that can be enhanced by a process called preconditioning. Preconditioning occurs when the cells undergo some modifications or are exposed to certain substances/environmental changes before being used for the aim of the experiment. For example, hypoxia is the most common form of precondition, and it has been shown that neural stem cells exposed to a hypoxic environment showed better survival, proliferation and differentiation. Usually, this happens via HIF-1 activation, as will be discussed further in the specific experiments. Another way to precondition the stem cells is genetically modifying them, although this has to be done with special care. Genetic modifications can also influence the basic genetic profile of the cells, making them unsuitable for in vivo usage. An example of genetic modifications is the overexpression of BDNF, GDNF and VEGF. Some cases of genetic modification in stem cells will soon be discussed [33].

Scientists tried to take advantage of the cellular environment that is created after a stroke, with the idea that the cells will find themselves in such an environment that cannot be ignored. Following this idea, tentative steps to “train” the cells to have a lower energy demand were made. Exposing tissues to intermittent ischemia makes them more adaptable to a state of oxygen and glucose deprivation (OGD) that could be found after a stroke. Since exposing a patient to a hypoxic environment could represent clinical harm, there was a need to find a way to induce the metabolic switch. If the environment has an abundance of glucose, glycolysis is the main pathway followed. In the case of glucose deprivation, the metabolism will rely on oxidative phosphorylation. Enhancing mitochondrial function is an approach that aims to reduce the area of dead cells around the insult, in the case of stroke, the core area. The cells chosen for this test were extracted from the umbilical cord (UC-MSCs), and they were cultured in normal conditions. For the metabolic switch, galactose was used to substitute glucose in the medium. The oxygen consumption rate/extracellular acidification rate (OCR/ECAR) ratio confirms that MSCs normally rely on glycolysis as a primary energy resource. Switching the metabolism from glucose to galactose increases the need for oxidative phosphorylation, increasing OCR/ECAR ratio. This evidence suggests the application of a specific diet to adjust the cellular metabolism of the patient during routine hospital feeding [34]. Using a similar approach, other authors found the development of so-called “super mitochondria” in MSCs grown in metabolic-switching environments. These organelles were able to produce more energy compared to the normal ones. The co-culture with neurons displayed a reduction in ROS production when exposed to metabolic-switched MSCs in oxygen-deprived conditions. In a similar way, the amount of ATP mRNA produced by mitochondria was restored in this latter-mentioned group [35]. The problem of energy supply for cells during lack of oxygen due to an impairment in blood flow has to be taken into consideration. As mentioned before, the excessive consumption of energy, compared to the amount that can effectively be used, can lead to neuronal death and toxicity, so the preparation of stem cells in a low-energy environment can boost their efficiency and survivability.

Interleukin-17A (IL-17A) was observed to peak twice after middle cerebral artery occlusion (MCAO) in mice, a practice used to model stroke in animals. IL-17A is secreted from astrocytes, thanks to the nuclear factor kappa-light-chain-enhancer of activated B cells (NF-κB) and, in turn, promotes the expression of BDNF, glial cell-derived neurotrophic factor (GDNF) and nerve growth factor (NGF), thus enhancing repair in brain tissue. IL-17A triggers mitogen-activated protein kinase 14 (p38) MAPK/calpain 1 signaling pathway in order to increase neuronal precursor cells’ survival and synapse formation. Another signal that proves to be relevant in the MCAO model is Wnt Family Member 3A (Wnt3a), which is reduced 24 h after surgery. Its presence can ameliorate the condition of the damaged area, restoring part of the tissue and thus improving neurobehavioral functions. The last signal that was taken into consideration is Notch because its inhibition can, in turn, inhibit neuronal apoptosis, preserving the remaining neurons. For this experiment, the authors used mesenchymal stromal cells derived from the bone marrow (BM-MSCs) of rats, and they were cultured with different concentrations of IL-17A for seven days. A successful expression of neuronal differentiation genes was obtained at low concentrations, while over 20 ng/mL no differential expression was found. Moreover, 40 ng/mL proved to increase neuronal death. The immunofluorescence analysis confirmed the presence of neuronal differentiation markers, as well as morphological analysis. The subsequent Western Blot analysis aimed to investigate the involvement of the Notch and Wnt pathway in the differentiation. The Notch pathway seemed inhibited while Wnt was upregulated, but if the inhibition of the Wnt pathway resulted in the blocking of the Notch signal, the other way around was not happening. This suggested a finer regulation of neuronal differentiation of BM-MSCs. Analyzing the molecular mechanism, it was found that the inhibitor of nuclear factor kappa-B kinase (IKKα/β) phosphorylation was downregulated, indicating that the NF-κB pathway was repressed. This increases the amount of Hes Family BHLH Transcription Factor 1 (Hes-1), one of Notch’s intracellular domains. The inducer of the Wnt pathway, enolase, was found to increase after IL-17A exposition [36]. This experiment indicates that BM-MSCs, using Notch/Wnt/NF-κB signaling, have the potential to efficiently differentiate into neurons when exposed to IL-17A, but the high concentration is detrimental. The presence of IL-17A in the brain area after stroke makes BM-MSCs a very good candidate for stem cell therapy, even if it will be interesting to investigate if another type of stem cell retains the same potential or, maybe, a different choice of stem cells could result in a faster or more efficient neuronal differentiation in neurons even faster or more efficiently.

This is the case of olfactory mucosae MSCs (OM-MSCs), which, if exposed to a hypoxic environment, show the capacity to differentiate in dopaminergic neurons. Olfactory mucosae represent a virtually never-ending source of MSCs, which are very well tolerated and resistant to mutations, and they have a high proliferating ratio. They seem, indeed, a good source of dopaminergic neurons due to their capacity to differentiate them if cultured in hypoxic conditions. In their study, authors cultured OM-MSCs in hypoxic conditions, and they confirmed their increased release of dopamine compared to the normoxic MSCs. Investigating the possible mechanism to explain this efficient change, HIF-1 was taken into consideration since it is a sensor of low amount of oxygen, and it is, of course, involved in the hypoxia response. HIF-1α upregulation seems to mediate the expression of genes and the protein amount involved in the dopaminergic development, such as LIM Homeobox Transcription Factor 1 Beta (Lmx1b), Paired Like Homeodomain 3 (Pitx3), nuclear receptor 4A2 (Nurr1), Engrailed Homeobox 1 (En1), and Engrailed Homeobox 2 (En2) [37]. Even though the main benefit from this particular treatment would be in Parkinson’s Disease, dopaminergic neurons could also be lost after stroke, so this evidence could be helpful in the possible treatment of different diseases.

Thanks to the development of iPSCs technology, new therapeutic approaches could be designed, and the possibilities of innovative treatments increased. The observation that stem cells reside within ischemic areas of patients after brain stroke, along with the discovery that they have a profile similar to MSCs, triggers the idea of a comparison between them in order to understand which one has the best therapeutic potential in stroke. The study compared ischemia-induced multipotent stem cells (iSCs) with BM-MSCs, and the neuronal differentiation potential of iSCs was tested. The two types of cells were cultured in adipogenic, osteogenic, or chondrogenic differentiation medium, while the neuronal differentiation potential was assessed using a neurobasal medium with basic fibroblast growth factor (bFGF) and B27 supplement. The important discovery in this experiment was that only iSCs could efficiently differentiate in neurons, while BM-MSCs have a better potential to differentiate in adipocytes, osteocytes, and chondrocytes. The neurons resulting from iSCs differentiation can also generate action potentials [38]. The development of this new type of stem cell represents a huge advancement for the scientific community and is also an example of how scientific research could be refined. This experiment is not in contrast with the previous one since the source of stem cells is quite different, but the comparison is useful to understand that not all stem cells are equal and they have very different potentialities. Their discovery will help catalog the stem cells according to the type of cells into which they better differentiate.

Table 1 represents the in vitro evidence of how preconditioning can be useful to enhance stem cell qualities.

## 6. Routes of Administration for Stem Cell Therapy

In vitro findings need to have their confirmation in vivo, where the conditions are more similar to the human brain compared to in vitro cultures. Another important challenge regarding regenerative medicine is represented not only by the correct choice of stem cell type but also by how they are administered to the patients. The different delivery systems result indeed in a diverse bioavailability of the stem cells, making the difference between an efficient reach of the target and a waste of materials.

The main model used for simulating the effect of human stroke is MCAO in rodents, such as mice or rats, but it is also used in big animals like pigs. MCAO consists of the surgical occlusion of the middle cerebral artery and stops blood flow. The surgery involves the insertion of a filament inside the external carotid artery, moved along the blood vessel to the internal carotid artery until the tip of the filament reaches the bifurcation of the middle cerebral artery, thus blocking the blood flow. This results in brain infarction around the area of occlusion, and it can be permanent or temporary, modeling a permanent MCAO or transient MCAO; the latter is also used to investigate the effect of reperfusion [39].

As for drugs and substances in general, stem cells can be infused in the model using different routes. The most commonly used is intravenous because of its easy access, and intracerebral, where cells are directly injected into the area of interest, but both of them have advantages and disadvantages. 

### 6.1. In Vivo Tests with Classical Routes of Administration: Optimizing Existing Techniques

The reduction of the inflammatory response in the ischemic area can be obtained by different approaches. With the aim to also decrease the side effects of synthetic anti-inflammatory products, scientists sometimes look at natural products. For example, royal jelly, a natural bee’s product with a wide range of beneficial effects, from immunoregulatory to antibacterial, also seems to be beneficial for memory. Using a combination of MSCs and royal jelly, the authors tested the therapeutic effects of MSCs in the MCAO mouse model. The cells were harvested from the bone marrow of femurs and tibias of eight-month-old mice and characterized for adherence and cell surface markers, as well as testing the multilineage differentiation. The royal jelly was suspended in water and injected intraperitoneally. The results indicate that royal jelly decreases the serum level of interferon-gamma (IFN-γ) as the Th1 cytokine, while treatment with MSCs reduces the brain level of IL-1β. However, MSCs seemed to increase the presence of Th1 cells in the infarcted area, while royal jelly increased instead Th2 population. It is theorized that royal jelly can, in some way, compensate for the long-term inflammation due to MSCs administration [40].

#### 6.1.1. Intracerebral Administration

Intracerebral infusion is another route of administration for stem cells, even though it is clear that the main disadvantage is the surgical procedure. Usually, the intracerebral infusion is done by stereotactic surgery, which requires trained scientists, and stereotactic apparatus and is not free from the risk of death for the animal. Briefly, the animal has to be anesthetized, and the skin over the head has to be cut and opened. The skull will be drilled over the point of infusion, which is defined by coordinates based on an atlas. Then, cells or substances are infused into the selected area after a very slow insertion of the needle into the brain through the skull hole. When the infusion is complete, and the needle is outside the brain, the skin can be closed, and the animal can be awakened [41]. Of course, this procedure also requires post-surgery monitoring, with analgesic administration and deep attention due to the presence of sutures, which can be opened by the animal itself or by companions in the same cage. The undoubted advantage is that this procedure bypasses the bloodstream and direct immediately the cells where the scientist wants them to settle since one-third of the injected cells usually migrate toward the damaged regions [42]. In addition, for humans, the procedure is quite invasive. Before the surgery, it is required to do a CT scan in order to standardize the intraoperative navigation, then under local anesthesia, the skull of the patient is drilled, and the catheter is implanted in the area of seeding, that could be the ventricles [43,44]. Intracerebral administration overcomes the blood flow problem, and it can deliver the cells directly where they are needed, but it is difficult to translate it into humans. The procedure requires a surgical operation and expert physicians, and probably not all the patients are suitable to undergo surgery, so it is not widely applicable for clinical practice. 

The hostile environment that the cells will encounter in the brain after stroke represents an issue for the survivability of MSCs. One of the strategies to implement survivability is the precondition, and activating HIF-1α seems to give promising results. BM-MSCs were extracted from rats and cultured in oxygen-deprived conditions. They were also characterized with flow cytometry, and their differentiating potential was assessed. It is important to mention that the cells were marked with Green Fluorescent Protein in order to be easily tracked into the brain. One selected group also received pre-treatment with FG-4592. The common name for this substance is Roxadustat, and it is known to activate the HIF-1α pathway, also stabilizing the level of HIF-1α. The animals underwent permanent middle cerebral artery occlusion (pMCAO) before being treated with BM-MSCs, injected directly into the brain. Results demonstrate that pre-treating BM-MSCs with FG-4592 increases their chance of surviving hypoxic-induced apoptosis. This happens probably through an enhancement of autophagy. Indeed, suppressing this process using 3-MA also reduces the anti-apoptotic effect of FG-4592 on BM-MSCs. In addition, the HIF-1α pathway can lead to autophagy, so FG-4592 can induce autophagy using the HIF-1α/BNIP3 signaling pathway. The advantage of BM-MSCs also lies in their site of origin because the bone marrow is already at a low oxygen condition, so HIF-1α is probably crucial for many aspects of BM-MSC survival and function. For this reason, there is no surprise to find that the pre-treatment with FG-4592 increases the survivability of BM-MSCs after transplant compared to the BM-MSCs alone [45]. This important evidence regarding the crucial role of HIF-1 is supported by the next described experiment of genetic modification using nanoparticles. Using neural stem cells taken from newborn mice, the authors tried to design nanoparticles for efficient delivery of a functional peptide into the cells in order to upregulate HIF-1-related pathways. The cells used, expressed a high level of nestin, and it is probable that they were chosen for their high potential of differentiating into the neuronal lineage. The previous main problem was that the upregulation of HIF-1 was obtained using viral vectors, which in turn disturbs the genetics of the cells and limits their in vivo application. To efficiently track NSCs after functional peptide delivery, the nanoparticles were loaded with superparamagnetic iron oxide nanoparticles (SPIO), so they could be tracked using MRI. The model used was different from MCAO because it was obtained using photothrombotic ischemia (PTI). The skull of the mice was opened, and the intraperitoneally injected dye, named Rose Bengal, was activated using a 50 mW of 532 nm green laser pointed to the targeted brain area for 15 min. The marked-NSCs were stereotactically injected into the infarct area. The delivery of the functional peptide was successfully achieved since the authors proved the increased amount of HIF-1α and CXCR4, a downstream target in the HIF-1 cascade. Casp8ap2, involved in the apoptotic signaling, was significantly decreased, indicating less tendency of the cells to die and improving their bioavailability. Transplanted mice revealed less volume of infarction one week post-surgery, while two weeks later, the ratio of apoptotic cells was inferior in NSCs treated with the functional peptide. Indeed, more NSCs positive for nestin and CXCR4 were found around the transplanted area, and they seemed to migrate efficiently to the cortex [46].

Pretreating the stem cells before transplant became a widely used technique. In the case of trehalose (Tre), authors used it as pretreatment for BM-MSCs before transplant. The cells were harvested from the tibiae and femur of 3-week-old rats and characterized with flow cytometry for membrane markers. The authors also characterized the cells after they were pre-treated with Tre in order to assess if the treatment changed their genetic profile. In vitro evidence already indicated that Tre is able to induce autophagy in BM-MSCs using the AMPK pathway, as the levels of LC3B indicated, and it is protective against oxidative stress by influencing the Bcl-2/Bax ratio. Autophagy proved to be important for clearing toxic protein aggregates, like in Parkinson’s Disease, via the mTOR pathway, so enhancing this process is protective for the cells. Tre-BM-MSCs secrete BDNF, VEGF and HGF, which are all protective for neurons, and indeed lesioned rats experience an improvement of the infarcted area after MCAO. Neuronal Nuclei (NeuN) staining confirmed the increased presence of neurons when treated with Tre-BM-MSCs. Tre-BM-MSCs were also beneficial for vascularization since they improved the microvessel density in the cortex and striatum area [47]. The secretion of beneficial factors, such as the mentioned BDNF, VEGF and HGF, appears to be one of the key features of BM-MSCs, making them an additional source of molecules that can trigger the physiological regeneration of the brain. The increased presence of neurons could be due to the stem cells in the neurogenesis niche, influenced by BM-MSCs paracrine effects.

Targeting specific genes and modifying their expression in stem cells used for therapy is a fascinating approach. It allows researchers to enhance some properties of the stem cells and prepare them for their tasks or allow them to influence the environment in a positive way in order to improve regeneration. It is the case of the Maudsley hippocampal murine neural progenitor line clone 36 (MHP36) cell line, a conditionally immortalized neural stem cell (NSCs) line. What is interesting about this cell line, derived from H-2Kb-tsA58 transgenic embryonic mouse hippocampal neuroepithelium, is that they can expand at the temperature of 33 °C. When put at 37 °C, which is around human, rat and mouse body temperature, they stop proliferating and start to differentiate. For this experiment, the RNA of the gene *Dax1* was degraded, and its protein amount was severely reduced by a short hairpin (sh) RNA targeted to *Dax-1.* The aim was to increase the production of 17β-estradiol, which has neuroprotective properties and beneficial effects on recovery in stem cell therapy after cerebral or myocardial ischemia. Since the pre-clinical evidence was not translated into humans, or even worse, the 17β-estradiol led to a worsening of the condition after stroke; it was necessary to take a step back and optimize the cellular condition before another clinical attempt. MHP36 cells were chosen because of their excellent behavior in repairing brain damaged tissue. They migrate to the site of injury and restore cognitive and functional activities without forming tumors. MHP36-Dax1KD, MHP36 cells with Dax1 knockdown, were intracerebrally injected in MCAO mice. The mice injected with the modified cell line have a better and faster recovery after transient stroke compared to controls. The crucial result is the complete recovery of the treated mice to functional levels before MCAO, as demonstrated by the cylinder and foot fault test. The observation that the mice tend to use the contralateral paw when treated with unmodified MHP36, or MHP36 cells suspended in 17β-estradiol, led to further investigations regarding synaptic plasticity. The results indicate that 17β-estradiol increases synaptogenesis, as suggested by MHP36-Dax1KD cells’ co-localization with Growth Associated Protein 43 (GAP-43), which is usually located in the axonal growth cone. Moreover, Microtubule Associated Protein 2 (MAP-2) presence indicates that the cells preferably differentiate into neurons than into astrocytes or oligodendrocytes since Glial Fibrillary Acidic Protein (GFAP) and 2′,3′-Cyclic-nucleotide 3′-phosphodiesterase (CNPase) staining indicate no significant changes between groups. The lesion size was significantly reduced even after 48 h, while normally, the intervention with 17β-estradiol is indicated at 6 h to exert the neuroprotective effects. The neuroprotective effect of the 17β-estradiol is also combined with a certain degree of differentiation, so there is no surprise to know that the human equivalent of MHP36 is currently tested in the clinic [48].

Transplanted cells can be encapsulated in order to improve their bioavailability. This can also enhance their properties. One type of encapsulation can be Chondroitin-4-sulfate (CS-A), which, in combination with neural progenitor cells (NPCs), can ameliorate vascularization and increase post-transplant survival of the cells. CS-A seems to have a high affinity with immunomodulatory factors, so it is important to evaluate its interaction with microglia/macrophages. The cells chosen for this experiment are mouse-induced pluripotent stem cell-derived neural progenitor cells (iPSC-NPCs), which were differentiated from iPSCs from mouse embryonic fibroblasts origin. GFP introduction allowed the tracking of the cells when injected. The model used is similar to MCAO, except for the fact that the stroke was modeled by ligation of three to six branches and major collaterals of the middle cerebral artery. Intracerebral infusion of CS-A+NPCs resulted in an increase of Monocyte chemoattractant protein-1 (MCP-1) in microglia, which is an important chemoattractant for recruiting vascular-associated macrophages. Indeed, animals treated with this combination display better angiogenesis and arteriogenesis. MCP-1 also has the function of recruiting macrophages which can help in neurogenesis. This was demonstrated by increased levels of ionized calcium-binding adapter molecule 1 (Iba1) positive cells, as well as the levels of Interleukin (IL-)10. It is important to notice that CS-A encapsulation promotes the recruitment of Peroxisome proliferator-activated receptor gamma (PPARγ)-expressing microglia/macrophages and increasing the level of IL-10. PPARγ-expressing microglia have a regenerative profile, while IL-10 seems to recruit pro-arteriogenic microglia; indeed, the CS-A increased Vascular endothelial growth factor receptor 2 (VEGFR2), Tyrosine-protein kinase receptor 2 (TIE2), and Fibroblast growth factor 2 (FGF2) microglia expression. Encapsulated NPCs improved the scores in behavioral tests such as tail suspension and open field, and the scores were correlated with the level of PPARγ and IL-10, suggesting their important influence on post-stroke depression [49].

Since inflammation represents an important source of danger for cells, and in particular for the transplanted ones, tentatively reducing the inflammatory environment can improve the outcome of MSC regenerative medicine. The inflammatory response after stroke is mediated by different signals, and one of these is represented by Toll-like Receptor 4 (TLR4). Its signals are repressed by miR-185-5p, so authors theorize that overexpression of miR-185-5p can block TLR4/NF-κB signaling and potentiate the beneficial effects of BM-MSCs. The cells were harvested from mouse bone marrow and characterized using flow cytometry, then transfected with an miR-182-5p mimic. The cells were transplanted in the MCAO mouse model with a stereotactic injection after ensuring that the suppression of TLR4 did not alter the identity of BM-MSCs+ miR-185-5p. The microglia were indeed less reactive, and this resulted in a reduction of IL-6, IL-1β e tumor necrosis factor-alpha (TNF-α). The TLR4/NF-κB pathway was not as functional as in the stroke model without BM-MSCs+ miR-185-5p application. These effects resulted in obtaining better scores in neurobehavioural tests from the mice transplanted with BM-MSCs, which was even higher in the BM-MSCs+ miR-185-5p group [50].

Another important and easy-access source of stem cells is dental pulp, and they are usually taken from the host. They originate from the neural crest and oral-derived epithelial stem cells, possessing the capacity to also differentiate into neuronal cells. It is important to notice that, even without any induction, dental pulp stem cells (DPSCs) express neuronal markers like nestin or β-III tubulin and can secrete neurotrophic factors.

Healthy human volunteers agreed to the extraction, from their third molar, of the cells necessary for the study. The dental pulp tissue was harvested from the third molar using a dental handpiece, then cut and digested before being put in culture. The cells were characterized by flow cytometry and their differentiating potential was assessed. Then, using a model of transient MCAO in rats, DPSCs were injected directly into the brain close to the infarcted area. Results demonstrated that the animals treated with DPSCs have a better outcome compared to the controls in terms of neurological functions, including sensation, reflex, balance and motion. This was probably due to a reduction of brain edema and cellular apoptosis, evaluated by Bcl-2/Bax ratio and caspase 3 expression, and TUNEL staining. Moreover, NeuN staining revealed an increased expression of this marker in treated animals which, together with previous results, confirmed the decreased number of apoptotic cells in DPSCs-treated animals [51].

The mentioned studies have some important messages to deliver. The type of stem cells chosen for the experiment does not seem very relevant for the outcome since all of them obtained significant results in the models; the pretreatment is very important; it can dramatically make the difference between a mediocre response and a good one. It is important to remember that all of these experiments are carried out using the intracerebral route of administration: even though it is efficient in delivering the stem cells directly into the brain, bypassing the majority of obstacles that can decrease the efficiency of the therapy, it is not the easiest way to administer therapy to a patient.

#### 6.1.2. Intravenous Administration

The starting point for the analysis of the intravenous administration of stem cells for stroke therapy is that the intracerebral one is not applicable to all patients. The easiest and less invasive route of administration, both for patients and doctors, is through the blood vessels.

Intravenous injection is the most convenient and immediate route of administration. The main advantage, along with the easy access, is that it can simulate very well what could happen in humans. Once MSCs are infused intravenously, they distribute in the body through the bloodstream and mainly remain in the lung, spleen, liver, bone marrow, thymus, kidney and skin and, of course, tumors [52]. As is understandable, intravenously injected stem cells tend to remain where the vascularization is higher, so maybe for this reason, there is no difference between healthy animals and disease models, apart from the obvious difference between oncologic models and wild-type animals. In addition, the origin of the cells, meaning if they are autologous/syngeneic, allogeneic, or even xenogenic, does not make any difference in terms of tissue distribution [53]. It is interesting to notice that the number of cells declines over time after infusion, and after seven days, they are basically undetectable [54]. This phenomenon cannot be attributed to the apoptosis of MSCs, but in part is due to the blood flow passing in the lungs.They have a peculiar vascularization system, with very small vessels and a huge capillary network, which work in combination with the adhesion property of MSCs and results in great engraftment of MSCs in that site. Another issue is the risk of embolism or calcium deposits due to osteogenic differentiation [53]. Intra-carotid administration can be used to bypass large organs; however, the doctor has to remember that the cells will inevitably be trapped in brain vasculature. Since blood vessels in the brain have a small diameter compared to the rest of the body, the cell size is crucial, as well as cell dose, in order to maintain the correct perfusion. The perfusion must be coordinated with the physiological one since a high infusion speed could lead to hemorrhage, and this can be assessed by an arteriogram. Imaging is an important part of cell delivery, which does not stop simply when the cells are physically injected into the bloodstream. The monitoring of the patient’s health after infusion remains crucial [55]. As for intravenous administration, only a small number of transplanted cells arrived at the desired area, between 1-10%. It was shown that intra-carotid injection induces better cellular migration and engraftment than the intravenous one. It is important to point out that, in the mentioned experiments, both intravenous and intra-carotid injected stem cells seemed to have the same beneficial effects on stroke recovery [56]. 

Intravenous administration of stem cells is strongly dependent on the bloodstream flow, and, as mentioned before, the injected cells suffer the risk of being trapped in vessels outside the area for which the cells were meant. In the case of stroke, where the brain should be the targeted area, blood circulation has to pass through different big organs before reaching the brain. Intra-carotid administration can bypass the large organs, but the problem of a low number of cells that reach the target compared to the number of injected ones remains. The problem does not seem to be the blood-brain barrier (BBB) since stem cells proved to cross it efficiently. BBB is formed by endothelial cells adherent to each other thanks to the tight junction and being surrounded by astrocytes. This forms a very selective barrier, and one of the main difficulties of the brain’s drug is indeed to cross the BBB. This does not seem to be the case for stem cells, which appear to cross it thanks to paracellular or transcellular pathways, even though it has to be remembered that a lot of brain diseases have the disruption or lack of BBB as a consequence. In addition, stem cells also seem to possess the ability to release the tight junction between endothelial cells [57]. The main stem cell route of administration, as mentioned, is usually intra-carotid. MSCs cultured in 2D have the important disadvantage of getting trapped in the lung, preventing the reach of different body sites.

OM-MSCs were harvested from healthy human volunteers and isolated from their OM. This was cut and cleaned from blood and other debris. Cells were injected intravenously in the MCAO rat model, with the aim of adapting them to the microenvironment of the penumbra zone after stroke, and this was obtained by ischemia-hypoxia protocol. Particular attention was dedicated to miR-181a, whose downregulation seems to protect neurons from death while enhancing the protective function of neuronal mitochondria. Pretreating MSCs enhance their capacity to reduce neuronal death and decrease ROS production. MiR-181a regulates heat shock protein 70 (HSP70), glucose-regulated protein 78 (GRP78), anti-apoptotic Bcl-2, and myeloid cell leukemia-1 (McL-1), which actions include regulating mitochondrial functions and neuroprotection. Indeed, in preconditioned MSCs, miR-181a is downregulated, allowing the overexpression of its downstream products with neuroprotective and mitochondrial preserving functions [58]. This approach is interesting and confirms the strong potential of MSCs to have neuroprotective functions; however, the neuroprotection and preservation of mitochondrial function could not be enough to reverse the effects of stroke.

Recent years have seen a rise in consideration regarding the so-called “gut-brain axis”. Once thought to be isolated in the intestine, the microbiota gained increased consideration through the observation that its composition can have important influences on the overall health of a person. Focusing on stroke consequences, signals from the hypothalamic-pituitary-adrenal axis are responsible for glucocorticoids and adrenocorticotropic hormone-releasing factor, which reduces gut motility and induces dysbiosis, the imbalance between the different bacteria population in the gut. Systemic infections after stroke are indeed the result of the disruption of the intestinal barrier, so the bacteria normally confined there are free to reach other organs. Stem cells were extracted from the bone marrow of the tibiae and femur of a four-week-old rat, and they were characterized using flow cytometry. BM-MSCs intravenously injected in MCAO rats efficiently increased short-chain fatty acid (SCFA)-producing bacteria. This information is important because of the role of this particular bacteria. SCFA is indeed used as a substrate in the metabolism of lipids, glucose and cholesterol, while it activates G protein-coupled receptors involved in the regulation of T cells. SCFA-producing bacteria also secrete mucus, which repairs the intestinal barriers through stimulation of tight junctions’ proteins. Moreover, the increased presence of *Lactobacillus* in BM-MSCs treated animals increased as well as the production of butyrate as an anti-inflammatory, protecting the damaged sites. All these properties of the mentioned bacteria are accompanied by an increase in the neurological functions of the animals after stroke and an important decrease of neuronal loss in the hippocampus, indicating how the brain and the gut are indeed strictly correlated [59]. A similar result was obtained in patients, where patients with acute ischemic stroke, with or without phlegm-heat syndrome, were compared to healthy controls, and their fecal bacteria were inspected. Results indicate that an alteration in richness and gut microbiota structure is present in patients with stroke compared to controls, which provokes more frequent gastrointestinal complications and is also associated with neurological problems. In detail, *Actinobacteria* and *Proteobacteria* seem to be most abundant after stroke, as well as *Verrucomicrobia* and *Synergistetes* [60]. 

Besides optimizing the already existent techniques, scientists also tried new stem cells from different sources in order to understand if the problems with BM-MSCs or MSCs, in general, can be overcome by simply changing the type of stem cells used. As mentioned before, in vitro evidence suggests that different types of stem cells could have different potentials, like the efficiency in differentiation. Using transient MCAO in rats, the aim was to understand if OM-MSCs injected intravenously could have a potential in the treatment of ischemia/reperfusion injury. If the stop of the blood flow is detrimental, so is the reperfusion consequent of recanalization. The ischemia/reperfusion injury is responsible for inflammation, oxidative stress and calcium overload, which are all elements that can lead to neuronal death. Particular attention was put on the Golgi apparatus, responsible for the so-called “GA stress” due to its involvement in oxidative stress. The GA stress response includes the calcium overflow, as well as the upregulation of Golgi Phosphoprotein 3 (GOLPH3) and downregulation of secretory pathway Ca^2+^-ATPase pump type 1 (SPCA1). GOLPH3 induces Golgi fragmentation and activates apoptosis when upregulated, while SPCA1 has the important role of calcium regulator. For these reasons, there was no surprise to find GOLPH3 upregulated and SPCA1 downregulated after MCAO in rats. Using OM-MSCs, isolated from the root of the medial aspect of the middle turbinate, the increased level of GOLPH3 was prevented, thus resulting in a mitigation of GA stress in the cells. The ability of OM-MSCs to secrete Pigment epithelium-derived factor (PEDF) was found to be crucial in the restoration of phosphatidylinositol-3-kinase/serin-threonin kinase 1/mammalian target of rapamycin (PI3K/Akt/mTOR) pathway, which resulted in being impaired in ischemia/reperfusion injury, and partially recovered when OM-MSCs were used. It is important to notice that knocking down PEDF in OM-MSCs results in an impaired restorative capacity, underscoring that the capacity to restore the PI3K/Akt/mTOR pathway relies on PEDF [61]. As for BM-MSCs, OM-MSCs retain the capacity to induce neuroprotection by the mitigation of GA stress. However, after the description of the previous experiment, it can be concluded that intravenous injection of stem cells seems to base the beneficial effect on the mitigation or control of the hostile environment after stroke, but the actual recovery of the brain function relies on the capacity of the brain to heal itself.

Table 2 represents the main findings for stem cell therapy in vivo using the classical route of administration and different precondition techniques.

### 6.2. In Vivo Tests with Non-Classical Routes of Administration: Intrathecal, Intraperitoneal and Intranasal Administration

#### 6.2.1. Intrathecal Administration

Intrathecal administration has been proposed as a method to deliver stem cells into the central nervous system in order to prevent the blockage of the cells. The intrathecal space is the space between the arachnoid mater and the pia mater. Besides the cranial nerve roots and blood vessels, it contains the cerebrospinal fluid (CSF) that will become the carrier for the cells [62]. The CSF would be responsible for carrying the cells to the site of damage, but one has to consider the tendency of MSCs to form aggregates. A blockage of CSF flow due to cell aggregates will have dramatic consequences for patients. The risk of aggregation of MSCs after intrathecal administration is a dangerous issue that has to be taken into consideration. A 3D cultured method for MSCs has been proposed in order to prevent the aggregation that could represent a risk factor for the use of MSCs in therapy. MSCs in 3D culture significantly reduce their volume by up to 70% and show a better differentiation potential and an increased secretion of beneficial factors. The reduced integrin expression allowed them to have less tendency to adhere to extracellular matrices. To translate the molecular results to an animal model, the authors used the intrathecal delivery route in MCAO rats to compare MSCs cultured in the 2D and 3D methods. The cells were harvested from the human placentae of healthy volunteers, characterized using flow cytometry and their differentiating capacity was assessed. As expected, 2D cultured MSCs tend to form aggregates more than 3D cultured ones, probably due to the higher expression of integrin α4 and Vascular cell adhesion protein 1 (VCAM1). VCAM1 block reduces ventricular enlargement. Integrin α4 and VCAM1 proved to be important in the mechanism of adhesion/aggregation of MSCs and have to be taken into consideration for intrathecal injection of MSCs [63].

Nanoparticles became a successful tool to deliver drugs whose bioavailability is difficult to enhance or cannot simply reach the target as efficiently as the experimenter wants. In this study, authors used nanoparticles intrathecally injected and enriched with Tanshinone IIA (Tan IIA) to improve the recovery of a stroke model of pigs treated with human induced pluripotent stem cell-derived neural stem cells (iNSC). Even though stem cells efficiently pass through the blood-brain barrier, this is not true for the majority of drugs, so intrathecal administration allows the cells to be distributed efficiently by CSF and avoids drug blockage from the blood-brain barrier. In this case, it allowed a decrease in the necessary dose of Tan IIA and a decrease in the frequency of administration. The iNSCs are derived from patients’ somatic cells and retain the possibility to differentiate in neurons, astrocytes and oligodendrocytes. They can also differentiate in GABAergic and glutamatergic neurons. Since Tan IIA is a potent antioxidant and anti-inflammatory agent, its combination with iNSCs might improve their engraftment and survivability after transplant in the inflammatory area of the brain after stroke. The stroke was modeled in pigs using the same MCAO protocol normally applied to rodents. The combination efficiently improves the post-stroke outcome by reducing tissue degradation as well as immune cell activation, clearly identified by a reduction of Iba1 positive cell activation. It also increases tissue recovery by inducing neurogenesis and neuroblasts migration, as proved by the increase of Doublecortin positive neuroblasts in the subventricular zone and lesion borders. The results compare pigs treated with the combination of TanIIA and iNSCS or treated with iNSCs alone. The mentioned results were possible thanks to Tan IIA action, which increases IL-4 and IL-13 levels and enhances cell survival by positively influencing the PI3K/Akt/mTOR pathway. NSCs seem to be a good tool to replace lost neurons due to their capacity to differentiate into at least two different types of neurons. The combination with TanIIA, which improves survivability and reduces inflammation, also tries to create a better environment for the stem cells attracted in the lesioned area from the neurogenesis niche [64]. The important limitation of this route of administration is the same as the intracranial one. the need for surgery limits the access to the therapy to a certain number of patients, excluding this route of administration from being a general one. Moreover, the risk of cell aggregation is added to the basic risk represented by the surgery, resulting in less efficiency and safety.

#### 6.2.2. Intraperitoneal Administration

Intraperitoneal injection as a route of administration is even easier to perform than intravenous one. However, the deposition of the cells in an area so far from the target organ, the brain, might represent a huge obstacle to efficiency maximization.

Intraperitoneal injection is probably the main route of administration of the majority of the substances in in vivo experiments. It allows easy access, fast administration and less stress for the animal since an expert scientist can do an intraperitoneal injection within seconds, reducing restraining time for the animal. Comparing intraperitoneal and intravenous injection of stem cells in a model of multiple sclerosis, it has been shown how intraperitoneal injection decreased inflammation in the brain by reducing leukocytes [65].

The authors used cells extracted from the inguinal fat of rats in order to culture AD-MSCs. The model was obtained through transient common carotid arteries occlusion (CCAO) because the aim was to investigate the effect of the reperfusion (96 h). AD-MSCs, administered 2 h after model induction, resulted in an increase of intracellular levels of AMP-activated protein Kinase alpha (AMPK-α) and Klotho-α. They protect the BBB from disruption and reduce oxidative stress as well as the inflammatory response after stroke. All of these processes are important in the degeneration and death of neurons, and since CCAO seems to specifically damage the hippocampus, memory and learning tests were administered to the animals. Non-treated rats cannot distinguish between a known and novel object, while AD-MSCs treatment restored this capacity. From a molecular point of view, transient CCAO increased the level of IL-6 and TNF-α in the hippocampal area, indicating an ongoing inflammatory process that could trigger apoptosis and also the disruption of the BBB, enhancing the dramatic consequences of stroke. AD-MSCs intraperitoneally administrated reduced the inflammatory markers, avoiding neuronal exposure to a dangerous environment and protecting neurons from apoptosis. Using the AMPK pathway, AD-MSCs increased the expression of the anti-apoptotic Bcl-2, and the expression of Bax, pro-apoptotic, was reduced. This results in a lower Bax/Bcl-2 ratio and a lower loss of neurons. Moreover, AMPK exerts its neuroprotection by reducing oxidative stress, inflammation, and consequentially apoptosis, while Klotho-α target TNF-α and IL-6, mitigating the inflammatory response [66].

Intraperitoneal injection was used for administering human placental mesenchymal stem cells (h-PMSCs) in the MCAO mouse model, with a special focus on angiotensin- converting enzyme-2 (ACE-2). It has been found that h-PMSCs express more ACE-2 than brain endothelial cells, and its inhibition prevents the ameliorating of neurological function. The infarcted area of the ACE-2-inhibited h-PMSCs was similar to the mice not treated with h-PMSCs. Cerebral perfusion was significantly decreased in mice treated with ACE-2-inhibited h-PMSCs, as well as sham and untreated mice. Despite this effect, authors were not able to find h-PMSCs in the brain of the mice, and their previous experiment indicated that intraperitoneal injection led to an accumulation of cells in the vascular compartment. Cells were, in any case, less than 1% of the total injected cells [67]. 

One study compared intravenous injection and intraperitoneal injection in terms of cell distribution in neonatal mice. The results support the evidence obtained in another study where radioactive-marked MSCs were injected intravenously and intraperitoneally into adult mice. The intravenous route primarily targets the lung and then, after 48 h, the liver. After intraperitoneal infusion, mainly the liver, spleen and kidneys were targeted, and then a small number of cells were found in the lungs [68]. For reaching the brain, neither the intravenous nor intraperitoneal route seemed optimal [69].

#### 6.2.3. Intranasal Administration

Intranasal administration of stem cells is quite a new technique, but it promises to give interesting results.

Intranasal administration is a route that was proposed as an alternative for intrathecal administration and, for stem cells, is relatively new. Intraperitoneal injection does not seem to allow the stem cells to arrive at the brain while they are using their immunomodulatory potential to reduce or control inflammation in the site of damage. It is true that the intrathecal route bypasses different barriers for cells and drugs, but in clinical practice, it is not easily applicable since it requires a surgical procedure. For these reasons, it was necessary to find a route of administration that has easy access, with direct connection to the brain without losing material in other organs and does not require surgical procedures so that virtually all types of patients can benefit from it. Indeed, intranasal administration of MSCs efficiently delivers the cells into the brain, probably using olfactory neural pathways and passing through the cribriform plate. It is interesting to note that this movement of cells can stand for hours or even days, demonstrating that migration is not due to the intranasal administration procedure itself but is something that happens physiologically [70]. This method can also be used in neonatal rodents, allowing easier manipulation and less risk of death [71].

HFSCs are located in the bulge area of adult hair follicles. They have many advantages, such as easy accessibility, abundance and plasticity, since they are derived from embryonic neural crests, and they can differentiate into neurons, glial cells but also osteocytes and melanocytes. Moreover, they are harvested from the host, so there is no danger of graft-versus-host disease. The intranasal administration has been tested in this study. Modeling stroke in rats using the MCAO technique, HFSCs administration proved to be efficient in reducing the infarcted area as well as ameliorating the score in the behavioral tests. These were possible, probably thanks to the restored level of NeuN and VEGF after cell transplantation, because MCAO had the effect of decreasing them. Moreover, MCAO increases the level of BDNF and Neurotrophin 3 (NT-3) in the cortex, while HFSCs administration manages to regulate the level of NT-3 [72].

As for other routes of administration, the intranasal one also requires optimization since it does not seem to be a stable method. However, it is attractive due to its great potential, so studies were performed in order to find the correct protocol. Pre-treatment was one of the potential solutions for enhancing cell properties, so authors incubated hNSCs in hypoxic conditions for 24 h. The cells were harvested from the cortex of aborted human embryos and characterized using flow cytometry. It is interesting to notice that hNSCs cultured in normoxic conditions cannot reach the brain when injected intranasally, probably because of their poor migration capacity, which stuck them into the nasal cavity, where they get cleared. The hypoxic cultured hNSCs have decreased apoptosis ratio, but it was clear that hNSCs cannot survive for a long time in nasal cavities, so multiple injections were used to overcome this problem. The position of the animal appeared important since the correct head angle allows the cells to remain in contact with nasal epithelium and not to be swallowed. The model used was the hypoxia ischemic model in neonatal rats. Pre-treatment with hypoxia enhances the expression of CXCR4, which allows the cells to have a greater migration potential than normoxic ones. Since only hypoxic-hNSCs can efficiently reach the brain, it was possible to track their pathway. It started from the olfactory epithelium and went into the tissue below the lamina propria after crossing the olfactory epithelium, then crossed the cribriform plate and reached the subarachnoid space. At this point, the cells used the subarachnoid space and the blood vessel to reach the injured area. The hNSCs never reached the parenchyma, at least not before 72 h, and they were located only in the ventral side of the brain. The success of transplantation was also reached thanks to microcatheter use and cell suspension volume change before administration [73]. As mentioned at the beginning of this study description, optimization was necessary and was obtained using the catheter. Future optimization might be focused on a better penetration of the stem cells into the brain parenchyma in order to increase efficiency and localize the stem cells directly where they are needed.

The migration increase was the aim of other experiments, where the authors tried to increase the migration and survival of stem cells after transplant using Insulin-like growth factor-1 (IGF-1) as pre-treatment, followed by OGD. Stem cells harvested from the bone marrow of rat femur were used and then underwent OGD protocol. The BM-MSCs were then intranasally injected in a mouse model of stroke, obtained by the permanent ligation of two to three branches of the middle cerebral artery, while the common carotid artery was only transiently blocked, and then reperfusion followed. In vitro, preliminary results demonstrated that IGF-1 could efficiently prevent apoptosis in BM-MSCs in OGD conditions. The activation of the IGF-1 receptor led to the activation of the PI3K/Akt pathway, which increases survival. It has to be mentioned that IGF-1 also increases the expression of CXCR4 on BM-MSCs surface, which is responsible for their migration capacity. In vivo, the treatment followed more or less the previous study with one more cell administration since the treatment was set for days three, five and seven after surgery. The combination of IGF-1 and BM-MSCs increased neurogenesis in the infarcted area and also promoted angiogenesis, induced by the action of IGF-1 on VEGF and Angiopoietin 1 (Ang-1), while neurogenesis appeared to be promoted by BDNF [74].

Another strategy is pre-treating MSCs with erythropoietin since it seems to ameliorate their paracrine effects. The model used was MCAO applied to very young rats in order to model the neonatal stroke. MSCs pre-exposed to erythropoietin, which was also intraperitoneally administrated in a separate cohort of animals. The refinement of the administration protocol was obtained using multiple administrations, the first three days after MCAO and the second seven days post-surgery. The results demonstrate that MSCs alone can efficiently ameliorate the sensorimotor capacity of the animals, but pre-treatment with erythropoietin also induces a sensible cognitive improvement that is long-lasting. It also noticed an increase in brain volume with both types of cells, pre-treated and not pre-treated, demonstrating that intranasal is an efficient route of administration for MSCs. However, the beneficial effects are not correlated with the differentiation of MSCs. Probably it was mainly due to the paracrine feature of the stem cells, enhanced by the pre-treating with erythropoietin [75].

The repeated intranasal injection of stem cells seems indeed a winning strategy to improve their potential in the treatment of stroke. The authors pre-treated BM-MSCs, isolated from the tibias of transgenic rats, in hypoxic conditions and modeled the stroke in adult mice. BM-MSCs were injected on days three, four, five and six after surgery. Thanks to a tracker, authors were able to see that BM-MSCs efficiently migrate to the peri-infracted region after 6 h and 24 h after the single administration. The amount of NeuN/ Bromodeoxyuridine (BrdU) positive cells in the peri-infarcted area demonstrates that BM-MSCs can efficiently increase neurogenesis. Glucose transporter 1 (Glut-1) is a marker of adult neural stem cells, and Glut-1/BrdU positive cells were increased too in the infarcted area. This happened 14 days after the stroke, while after 21 days, it was possible to notice an increase in cerebral blood flow, also indicating the angiogenic properties of BM-MSCs. Concluding with the behavioral tests, mice receiving the cells showed an improved sensorimotor score in the tests, in line with the increased neurogenesis and angiogenesis observed [76].

To better visualize the impact of transient MCAO and consequently the effect of MSCs intranasally administed, MRI was also used to understand the treatment effect on the white matter. Authors induced stroke in very young rats and injected rats intranasally with MSCs 72 h after surgery. This experiment, different from the previous ones, uses only one MSCs administration. Interestingly, MRI revealed that MSCs treatment was able to ameliorate white matter condition and sensorimotor functionality even one month after surgery. The fractional anisotropy was increased in the injured area, demonstrating a delay in white matter development [77]. Fractional anisotropy is a measurement of the movement of water molecules called “isotropic”, like CSF, usually valued at 0, and the movement of water molecules called “anisotropic”, like fiber bundles, valued at 1. In the first case, the water is free to move and diffuse in any direction, while in the second case, the water changes its pattern due to obstacles. In this case, the alteration of fractional anisotropy can indicate an alteration in myelin structure [78]. In the injured area, there was increased astrogliosis, as suggested by the amount of Glial fibrillary acidic protein (GFAP) positive cells compared to the contralateral side of the brain, which does not receive any injury. The administration of MSCs was able, 28 days after the injury, to increase axonal presence in the lesioned area, demonstrated by the presence of Neurofilament Heavy Chain (NHF). Moreover, MSCs administration decreases the lateralizing behavior, defined as the tendency of the animal to use preferentially one paw on the other [77]. 

Table 3 illustrates the non-classical route of administration and the effect of the injected cells on the different models.

## 7. Clinical Evidence

Despite all the efforts from pre-clinical studies to optimize existing techniques or to find new administration routes or a new source of stem cells, there is currently no established clinical practice for stem cell therapy.

On the website ClinicalTrial.gov, searching for “mscs” and “stroke” terms indicates 37 studies completed, from which only three have published results. 

The study NCT02448641 used intracranial administration of SB623 cells, which are the property of SanBio and are adult bone marrow-derived mesenchymal stem cells that underwent temporary genetic modification. The patients selected are the ones who experienced motor deficits after an ischemic stroke. Unfortunately, according to the results, the responder to the therapy does not seem so high in number compared to the non-responders. However, elaboration of results from the Sponsor is required in order to have a complete view of the outcome of the clinical trial [78].

In the study, NCT03004976 umbilical cord blood was infused intravenously into adult patients with ischemic stroke. Umbilical cord blood is rich with blood stem cells, and the protocol wanted the umbilical cord blood to be infused as early as three days after stroke and not more than 10 days. The patients were screened at different time points in order to assess the safety of the procedure, the improvement of neurological symptoms, and the quality of life. To date, the Sponsor did not provide an elaboration of the published results [79].

The last study with published results, NCT00859014, uses mononuclear cells infused intravenously in adult patients who experienced a recent stroke so within 24-72 h. Mononuclear cells are rich in stem cells and can be rapidly isolated from bone marrow, so they represent an attractive source of cells for stem therapy. From a safety point of view, the treatment was safe, but it required a lot of effort from different teams of the hospital due to the autologous harvest of bone marrow. Overall the 10 patients showed an improvement in their condition, even though it was not possible to compare them to another study where t-PA was administered alone, so this strategy can be feasible for patients with ischemic stroke in the 24-72 h window [80].

It is worth mentioning another study, registered under the code NCT03356821, that was done in neonates who experienced a perinatal arterial ischemic stroke (PAIS). Ten neonates underwent BM-MSCs intranasal administration, and since this was a first-in-human trial, it was important to evaluate the safety and feasibility of this procedure. Three months after administration, no serious adverse events were reported. MRI imaging indicates that the administration is feasible within seven days from symptom onset, and the treatment is safe for the first three months of life. As for therapeutic outcomes, such as motor dysfunction or neurological disorder improvement, they were not possible to assess [81].

Several limitations are slowing the progress of translating stem cell therapy into clinical practice. The mechanism of action is not completely clear, so it is difficult to design tailored therapy around the patient. The time window in which the stem cells could be infused and still be efficient is difficult to find, starting from the evidence that it is not so easy to identify the exact moment when the stroke happened [82]. As can be seen, by the clinical trials described before, one important missing factor is a standardized protocol for stem cell therapy, which makes it difficult to make the right adjustments.

**Figure 2:**
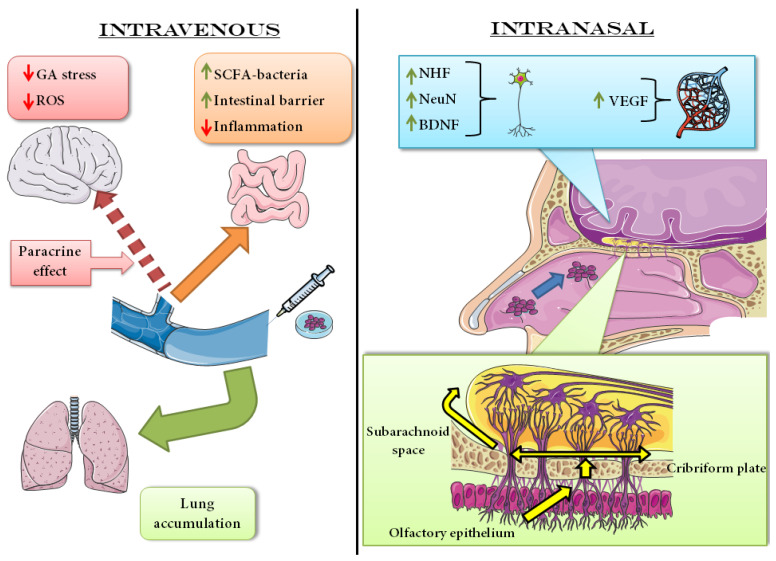
Schematic representation of the stem cells’ effects when injected intravenously or intranasally. In the first case, the cells tend to accumulate in the lungs, but they are able to exert their effect on other organs, such as the intestine and brain. Intranasal administration allows the cells to cross the olfactory epithelium, the cribriform plate, and then into the subarachnoid space, where the CSF will distribute the cells across the brain. In this situation, the stem cells increase the neurogenesis and the angiogenesis, obtaining the double effect of neuron recovery and blood flow restoration.

Figure 2 resumes the fate and the effect of the stem cells using two different administration routes.Intravenous was chosen because it is one of the most used and is easily translated into clinical practice, and intranasal because of its undoubted potential due to the direct delivery of the cells into the brain.

## 8. Conclusions

Stem cells, and in particular BM-MSCs, proved to be promising for stroke therapy. Between the different routes of administration for stem cell therapy, intranasal administration, due to the bypass of large organs and the bloodstream, is an efficient method to deliver stem cells into the brain, and it is worthy of further investigation, representing an easily accessible, fast and efficient route of administration for humans. It is reasonable to think that a combination of hypoxic pre-treatment and the use of BM-MSCs intranasally administrated will give surprising results in the advance of stroke therapy.

## Figures and Tables

**Table 1:** Different preconditioning strategies to increase the beneficial potential of stem cells.

Cells Precondition	Cell Type	Effects	Mechanisms of Action	Ref.
IL-17A exposure	BM-MSCs	Neuronal differentiation	IKKα/β phosphorylation decrease Hes-1 increase Enolase increase Notch/Wnt/Nf-κB partway induction	[36]
Metabolic switch	UC-MSCs	OCR/ECAR increase	Oxidative phosphorylation as a primary source of energy	[34]
Metabolic switch	MSCs	“Super mitochondria” development	ROS reduction Energy production increase	[35]
Hypoxia	OM-MSCs	Increased dopamine release Promote dopaminergic development	HIF-1α upregulation Expression of Lmx1b, Pixt3, Nurr1, En1 and En2	[37]
None	iSCs + BM-MSCs	iSCs can better differentiate into neurons	Not applicable	[38]

BM: bone marrow; Hes-1: Hes Family BHLH Transcription Factor 1; HIF-1α: Hypoxia-inducible factor 1-alpha; IKKα/β: Inhibitor of nuclear factor kappa-B kinase; IL: interleukin; iSCs: ischemia-induced multipotent stem cells; MSCs: mesenchymal stromal cells; OCR/ECAR: oxygen consumption rate/extracellular acidification rate; OM: olfactory mucosae; ROS: reactive oxygen species; UC: umbilical cord.

**Table 2:** Classical route of administration and preconditioning strategies in different animal models of stroke.

Route	Cells Precondition	Cell Type	Experimental Model	Effects	Mechanisms of Action	Ref.
i.c.	Hypoxia + FG-4592	BM-MSC	pMCAO	Enhancement of autophagy Increased survivability	HIF-1α/BNIP3 signaling pathway activation	[45]
i.c.	Trehalose	BM-MSCs	In vitro + MCAO	Increased autophagy Neuroprotection New vascularization	AMPK pathway induction Oxidative stress protection Bcl-2/Bax ratio increased Activation mTOR pathway BDNF, VEGF and HGF secretion	[47]
i.c.	Dax1 KD	MHP36	MCAO	Better and faster recover after model induction Reduction of the lesion site	17β-estradiol increased production Synaptogenesis increase GAP-43 and MAP-2 expression	[48]
i.v.	Hypoxia	OM-MSCs	In vitro + MCAO	Downregulation of miR-181a	Neuronal mitochondria protective function enhancement ROS decrease Regulation of HSP70, GRP78, Bcl-2 and Mcl-1	[58]
i.c.	CS-A encapsulation	NPCs	MCAO (3–6 ligations)	Increased angiogenesis Increased neurogenesis Score improvement in behavioral tests	MCP-1 increase in microglia PPARγ-expressing microglia recruitment IL-10 levels increase VEGFR2, TIE2 and FGF2 increase expression in microglia	[49]
i.c.	Nanoparticles (HIF-1α upregulation)	NSCs	PTI	Infarction volume reduction Efficient migration to the infarcted area	Casp8ap2 decrease Increased HIF-1α Increased CXCR4	[46]
i.c.	miR-185-5p overexpression	BM-MSCs	MCAO	Better scores in behavioral tests Less reactive microglia	TLR4/Nf-κB signaling block IL-6, IL-1β and TNF-α reduction	[50]
Not specified	None	MSCs + Royal jelly	MCAO	Compensation of long-term inflammation	IFN-γ serum level decrease IL-1β brain level decrease Th1 and Th2 presence increase	[40]
i.v.	None	BM-MSCs	MCAO	Reduced intestinal damage Reduced inflammation Mitigate stroke effect on microbiota	SCFA-producing bacteria population increase Stimulation of tight-junction proteins Butyrate production	[59]
i.v.	None	OM-MSCs	MCAO	Reduction of GA stress Restoring of PI3K/Akt/mTOR pathway	PEDF secretion GOLPH3 level reduction	[61]
i.c.	None	DPSCs	tMCAO	Better outcome for neurological functions	Brain edema reduction Bcl-2/Bax ratio increase NeuN increased expression	[51]

AMPK: AMP-activated protein kinase; Bax: BCL2 Associated X: Apoptosis Regulator; BDNF: brain derived neurotrophic factor; Bcl-2: Bcl2 apoptosis regulator; BM: bone marrow; BNIP3: Bcl2 interacting protein 3; CS-A: Chondroitin-4-sulfate; CXCR4: C-X-C chemokine receptor type 4; DPSCs: dental pulp stem cells; FGF2: Fibroblast growth factor 2; GA: Golgi apparatus; GAP-43: Growth Associated Protein 43; GRP78: glucose regulated protein 78; GOLPH3: Golgi Phosphoprotein 3; HGF: hepatocyte growth factor; HIF-1α: Hypoxia-inducible factor 1-alpha; HSP70: heat shock protein 70; i.c.: intracranial; IFN-γ: interferon gamma; IL: interleukin; i.v.: intravenous; KD: knock down; MAP-2: Microtubule Associated Protein 2; MCAO: middle cerebral artery occlusion; McL-1: myeloid cell leukemia-1; MCP-1 Monocyte chemoattractant protein-1; MSCs: mesenchymal stromal cells; NeuN: neuronal nuclei; NSCs: neuronal stem cells; NPCs: neural progenitor cells; OM: olfactory mucosae; PEDF: Pigment epithelium-derived factor; PI3K/Akt/mTOR: phosphatidylinositol-3-kinase/serin-threonin kinase 1/mammalian target of rapamycin; PPAR-γ: Peroxisome proliferator activated receptor γ; PTI: photothrombotic ischemia; Th-: T-helper; TIE2: Angiopoietin-1 receptor; TLR4: Toll-like receptor 4; TNF-α: tumor necrosis factor alpha; ROS: reactive oxygen species; SCFA: short-chain fatty acid; VEGF: Vascular endothelial growth factor.

**Table 3:** Non-classical route of administration and preconditioning strategies in different animal models of stroke.

Route	Cells Precondition	Cell Type	Experimental Model	Effects	Mechanisms of Action	Ref.
i.t.	3D culture	MSCs	In vitro + MCAO	Better differentiation potential Increase secretion of beneficial factors Fewer aggregates	Decrease expression of VCAM and integrin α4	[63]
i.t.	Nanoparticles (Tan IIA)	iNSC	MCAO	Reduction of tissue degradation Induction of neurogenesis Enhanced survivability	Iba1+ cells activation reduction Doublecortin+ neuroblasts increase in SVZ and lesion borders IL-4 and IL-13 increase PI3K/Akt/mTOR pathway enhancement	[64]
i.p.	None	AD-MSCs	CCAO	Reduced oxidative stress BBB protection Reduction of inflammation	IL-6 and TNF-α reduction Bax/Bcl-2 ratio decrease AMPK action on oxidative stress Klotho-α action on inflammation	[66]
i.p.	None	h-PMSCs	MCAO	Improved neurological functions	ACE-2 increased expression	[67]
i.p and i.v.	None	MSCs	wt	i.v. Primarily targets lungs i.p Primarily targets liver, spleen and kidneys	Not applicable	[69]
i.n.	None	HFSCs	MCAO	Reduction infarcted area Better scores in behavioral tests	NeuN e VEGF level restoration NT-3 level regulated	[72]
i.n.	Hypoxia	hNSCs	Neonatal HI	Brain engraftment Better migration	CXCR4 enhancement Apoptosis ratio decrease	[73]
i.n.	Erythropoietin	MSCs	MCAO	Ameliorate sensorimotor capacity and long-lasting cognitive improve	Increased brain volume Paracrine effects	[75]
i.n.	IGF-1	BM-MSCs	In vitro + pMCAO (2-3 branches) + tCCAO	Prevention of apoptosis Better migration Increased neurogenesis Angiogenesis induction	PI3K/Akt pathway activation CXCR4 increased expression VEGF, Ang-1 and BDNF secretion	[74]
i.n.	Hypoxia	BM-MSCs	pMCAO (2-3 branches) + tCCAO	Increased neurogenesis Sensorimotor score improved Increase cerebral blood flow	NeuN/BrdU positive cells amount increased Glut-1/BrdU positive cells amount increased Angiogenesis increased	[76]
i.n.	None	MSCs	MCAO	Improvement in white matter condition Improved sensorimotor functionality Decreased lateralizing behavior	NHF increased in the lesioned area	[77]

ACE2: angiotensin-converting enzyme 2; AD: adipose derived; AMPK: AMP-activated protein kinase; Ang-1: Angiopoietin 1; BDNF: brain derived neurotrophic factor; Bcl-2: Bcl2 apoptosis regulator; BM: bone marrow; BrdU: Bromodeoxyuridine; CCAO: common carotid arteries occlusion; CXCR4: C-X-C chemokine receptor type 4; GDNF: Glial derived neurotrophic factor; Glut-1: Glucose transporter type 1; HFSCs: hair follicles stem cells; HI: hypoxia ischemia; h-PMSCs: human placenta mesenchymal stromal cells; Iba1: ionized calcium-binding adapter molecule 1; IGF-1: insulin-like growth factor; IL: interleukin; i.n: intranasal; i.t: intrathecal; i.p.: intraperitoneal; i.v.: intravenous; iNSCs: induced pluripotent stem cell derived neural stem cells; MCAO: middle cerebral artery occlusion; MSCs: mesenchymal stromal cells; NeuN: neuronal nuclei; NHF: neurofilament heavy chain; NSCs: neuronal stem cells; NT-3: neurotrophin 3; PI3K/Akt/mTOR: phosphatidylinositol-3-kinase/serin-threonin kinase 1/mammalian target of rapamycin; Tan IIA: Tanshinone IIA; TNF-α: tumor necrosis factor alpha; SVZ: subventricular zone; VCAM: Vascular Cell Adhesion Molecule 1; VEGF: Vascular endothelial growth factor; wt: wild type.

## Data Availability

Not applicable.
